# Combined over-expression of two cytochrome P450 genes exacerbates the fitness cost of pyrethroid resistance in the major African malaria vector *Anopheles funestus*

**DOI:** 10.1016/j.pestbp.2021.104772

**Published:** 2021-03

**Authors:** Magellan Tchouakui, Leon M.J. Mugenzi, Murielle J. Wondji, Micareme Tchoupo, Flobert Njiokou, Charles S. Wondji

**Affiliations:** a LSTM Research Unit, Centre for Research in Infectious Diseases (CRID), P.O. BOX 13591, Yaoundé, Cameroon; b Parasitology and Ecology Laboratory, Department of Animal Biology and Physiology, Faculty of Science, P.O. Box 812, University of Yaoundé 1, Yaoundé, Cameroon; c Department of Vector Biology, Liverpool School of Tropical Medicine, Pembroke Place, L35QA Liverpool, UK; d Department of Biochemistry and Molecular Biology, Faculty of Science, University of Buea, P.O. Box 63, Buea, Cameroon

**Keywords:** Malaria, *Anopheles funestus*, Vector control, Metabolic resistance, Cytochrome P450, Fitness cost

## Abstract

Metabolic resistance driven by multiple P450 genes is worsening insecticide resistance in malaria vectors. However, it remains unclear whether such multiple over-expression imposes an additive fitness cost in the vectors. Here, we showed that two highly over-expressed P450 genes (*CYP6P9a* and *CYP6P9b*) combine to impose additive fitness costs in pyrethroid-resistant *Anopheles funestus*. Genotyping of the *CYP6P9b* resistance allele in hybrid mosquitoes from a pyrethroid-resistant FUMOZ-R and the susceptible FANG strains revealed that this gene imposes a fitness cost in resistant mosquitoes similar to *CYP6P9a*. Homozygote susceptible CYP6P9b_S (SS) significantly lay more eggs than the resistant (OR = 2.2, *P* = 0.04) and with greater hatching rate (*p* < 0.04). Homozygote resistant larvae CYP6P9b_R (RR) developed significantly slower than homozygote susceptible from L1-L4 (χ^*2*^ = 7.2; *P* = 0.007) with a late pupation observed for RR compared to both heterozygotes and homozygotes susceptible (*χ*^*2*^ = 11.17; *P* = 0.0008). No difference was observed between genotypes for adult longevity with no change in allele frequency and gene expression across the lifespan. Furthermore, we established that *CYP6P9b* combines with *CYP6P9a* to additively exacerbate the fitness cost of pyrethroid resistance with a greater reduction in fecundity/fertility and increased developmental time of double homozygote resistant mosquitoes. Moreover, an increased proportion of double homozygote susceptible individuals was noted over 10 generations in the insecticide-free environment (χ^*2*^ = 6.3; *P* = 0.01) suggesting a reversal to susceptibility in the absence of selection. Such greater fitness cost imposed by multiple P450 genes shows that resistance management strategy based on rotation could help slow the spread of resistance.

## Highlights

• Metabolic resistance to insecticide is major challenge in mosquito control.
• Fitness cost of metabolic resistance is poorly characterized.
• This is the first study evaluating the fitness cost of duplicated P450 using molecular marker.
• We showed that CYP6P9b combines with CYP6P9a to exacerbate the fitness cost of pyrethroid resistance in *Anopheles funestus*.

## Graphical abstract

**Figure:**
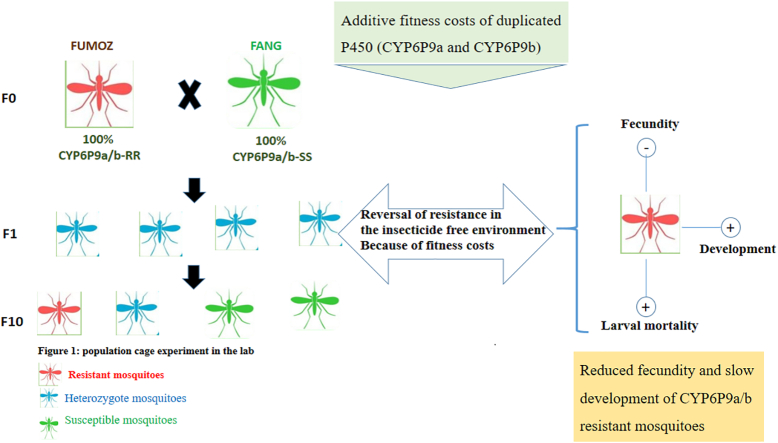

## 1 Background

Malaria burden remains high in Africa despite recent progress achieved mainly through insecticide-based interventions (long-lasting insecticidal nets (LLINs) and Indoor Residual Spraying (IRS)) (Bhatt et al., 2015; WHO. World Malaria Report, 2016). Increasing reports of resistance to major insecticide classes is a worrying concern for the continued effectiveness of insecticide-based control tools. Resistance to pyrethroids is particularly problematic, as it is the main insecticide class approved for LLINs impregnation, as well as the most common insecticide class used in IRS (Hemingway et al., 2013). To sustain the effectiveness of these interventions it is imperative to implement suitable insecticide resistance management (IRM) strategies to reduce the negative impact of such resistance. IRM strategies such as rotation of insecticide classes rely on resistance having a fitness cost causing selection against resistance alleles in the absence of insecticide selection pressure. Therefore, understanding the fitness cost that resistance imposes on the mosquito population is a key prerequisite to effective IRM as it could contribute to implement suitable control measures for malaria prevention.

Resistance can arise primarily through target-site resistance preventing the insecticides to reach the target or metabolic resistance which allows mosquitoes to digest the chemicals and detoxify them before they reach their target (Coetzee and Koekemoer, 2013; Ranson et al., 2011). Metabolic resistance is driven mainly through three enzyme families; cytochrome P450s, glutathione S-transferases, and esterases. If the specific genes driving metabolic resistance have been previously detected in major malaria vectors (Riveron et al., 2018), the underlying genetic variants driving this resistance mechanism are just beginning to be deciphered (Riveron et al., 2014). The design of the DNA-based diagnostic tools for target-site resistance since the late 1990s (Martinez-Torres et al., 1998; Ranson et al., 2000) allowed to study the fitness cost of target-site resistance on different life-traits in a range of mosquitoes species (Alout et al., 2016; Alout et al., 2014; Assogba et al., 2015; Brito et al., 2013; Martins et al., 2012a). However, fitness cost associated with metabolic resistance, a very common resistance mechanism (Hemingway, 2014), remains poorly characterized. Nevertheless, some key markers of metabolic resistance have been detected for glutathione s-transferase-mediated resistance: the L119F-GSTe2 marker in *An. funestus* (Riveron et al., 2014) and the I114T-GSTe2 in *An. gambiae* (Mitchell et al., 2014). A recent study evaluating the fitness cost of GST-based metabolic resistance in *An. funestus* using the L119F-GSTe2 marker revealed significant cost in the GST-resistant mosquitoes in terms of fecundity and larval development but a benefit regarding female longevity (Riveron et al., 2018) and vectorial capacity (Fadel et al., 2019). Such negative effects of L119F-GSTe2 mediated metabolic resistance on some life-traits of *An. funestus* field mosquitoes support the assumption that insecticide resistance is associated with a fitness cost. This shows that resistance management strategies such as insecticide rotation could help reverse resistance if implemented early.

Recently, significant progress was also made in detecting molecular marker for cytochrome P450 based resistance with the detection of cis-regulatory variants driving the expression of the duplicated pyrethroid resistance genes *CYP6P9a* and *CYP6P9b* in *An. funestus* (Weedall et al., 2019; Mugenzi et al., 2019). Two novel PCR diagnostic assays were designed to detect and monitor these mechanisms as well as assessing the fitness cost of P450-mediated metabolic resistance in the major African malaria vector *An. funestus (*Weedall et al., 2019; Mugenzi et al., 2019). A recent study assessing the fitness cost of *CYP6P9a* only, revealed that the resistant allele for this marker negatively impacted the fecundity and development time of resistant mosquitoes (Tchouakui et al., 2020a). In the same study, a significant decrease in the resistant *CYP6P9a*-RR genotype was observed in the absence of selection (Tchouakui et al., 2020b). However, the fitness cost imposed by the other over-expressed P450 as well as the over-expression of multiple pyrethroid resistance P450s remains unclear. The over-expression of detoxification genes such as cytochrome P450s is a process that is very demanding in energy for the mosquitoes and is known to take away resources and energy needed by the mosquito for growth, reproduction and other functions (Rivero et al., 2010). Thus, it could be expected that the over-expression of two or more of these genes simultaneously, as it is the case for *CYP6P9a* and *CYP6P9b* which have been shown to be highly expressed in some populations of *An. funestus*, could have additive fitness cost effect. However due to the previously lack of molecular markers for these P450s, such hypothesis could not be investigated. The recent design of the *CYP6P9b* DNA-based diagnostic tool, now allows not only to assess its own fitness cost but more importantly to establish whether up-regulation of multiple P450 conferring pyrethroid resistance leads to an aggravation of the fitness cost of pyrethroid resistance. A worsening of the effect caused by the combined expression of these P450s was recently observed for the ability to withstand exposure to insecticides as mosquitoes homozygote resistant to both genes significantly survive more after exposure to insecticide-treated nets than double homozygotes susceptible ones (Mugenzi et al., 2019). Thus, it is important to explore the fitness costs associated with over-expression of multiple cytochrome P450s and evaluate also the cumulative effect of these genes to better inform malaria control programs.

Here, we established firstly the fitness cost associated with *CYP6P9b* gene on the life traits of *An. funestus* and then the cumulative effect with the other P450, *CYP6P9a*. Furthermore, a reversal to susceptibility was evaluated in the absence of insecticide selection pressure using cage experiment approach.

## 2 Methods

### 2.1 Mosquito's strains

The same mosquitoes previously used for the assessment of the fitness cost associated with *CYP6P9a* (Tchouakui et al., 2020c) were used in this study to firstly evaluate the fitness cost linked with *CYP6P9b* and then the cumulative effect of *CYP6P9b* and *CYP6P9a*. These mosquitoes are from a hybrid strain obtained from the crossing between two *Anopheles funestus* laboratory strains: FANG (susceptible) and FUMOZ-R (resistant). Moreover, DNA from mosquitoes maintained in the insectary for 10 generations was used to genotype the *CYP6P9b* as previously done for *CYP6P9a* (Tchouakui et al., 2020c) to evaluate the reversal to susceptibility by monitoring the frequency of *CYP6P9b*-R resistant allele and combined genotypes from both genes over generations.

### 2.2 Life trait experiments

All parameters were evaluated by simultaneously comparing fitness parameters between homozygotes resistant, heterozygotes and homozygote susceptible mosquitoes for *CYP6P9b* and for both markers. We mainly focused on fecundity/fertility of females, time of larval development and adult longevity.

The impact of *CYP6P9b*-R resistant allele on fecundity was assessed by comparing the egg-laying ability, the median number of eggs laid and the hatch rate between different genotypes.

After recording the total number of larvae produced per female, all larvae from the three genotypes for each marker were pooled and reared together in the same larvae bowl to avoid variations due to environmental conditions. Changes in the time of development of immature stage and mortality rates were equally assessed by genotyping about 100 larvae at different stages (L1, L2, L3, and L4). For this purpose, genotype frequencies were monitored in each stage of development. The dynamic of pupae formation was evaluated by comparing the genotype and allele frequencies from the starting of pupation (pupae D9), in the third day of pupation (pupae D11) and on the fifth day of pupation (pupae D13).

After the emergence of adults, a set of about 150 mosquitoes was removed from the cages at different time points (day 1, 10, 20 and 30 after emergence). On average, 100 mosquitoes were used for genotyping whereas 3 pools of 10 mosquitoes each were used to assess the gene expression level of *CYP6P9b* at each time point. The lifespan of homozygous resistant adult mosquitoes was compared to that of susceptible and heterozygotes mosquitoes by assessing the frequency of *CYP6P9b* genotypes/alleles and the expression level of *CYP6P9a/b* (qRT-PCR) at different time points.

### 2.3 Population cage experiments to assess a potential reversal to susceptibility

The dynamics of *CYP6P9b*-R resistant allele frequency in the absence of insecticide pressure was assessed by cage experiments. After crosses between female FANG and male FUMOZ, the progeny obtained were let in cages for intercrosses for ten generations. In each generation, all mosquitoes irrespective of their genotypes were mixed in cages for intercrossing to generate the next generation. Each generation consisted of about 3 cages of at least 200 mosquitoes/cage of all genotypes. In the first generation, the frequency of the *CYP6P9b_R* resistant allele was assessed and then monitored in the following generations by genotyping a set of females aged between 2 and 5 days old.

### 2.4 Genotyping of resistance markers

Genomic DNA was extracted from adult mosquitoes and all larval and pupal stages using the LIVAK method (Livak, 1984). The genotyping of *CYP6P9a* resistance allele was done using PCR restriction fragment length polymorphism (RFLP) (using a Taq1 restriction enzyme) method as recently described (Weedall et al., 2019). *CYP6P9b*-mediated resistance was also genotyped by PCR-RFLP as recently described (Mugenzi et al., 2019). The restriction enzyme for this marker was NmuCl (*Tsp*45I) (cut site 5′-GTSAC-3′). This restriction enzyme cuts the susceptible mosquitoes in two fragments of 400 bp and 150 bp whereas the resistant is uncut at 550 bp.

### 2.5 Expression profile of *CYP6P9a* and *CYP6P9b* and adult longevity using qRT-PCR

Total RNA from three biological replicates (ten mosquitoes each) from D1, D10, D20, and D30 after adult emergence was extracted using the Picopure RNA Isolation Kit (Arcturus). The transcription patterns of the duplicated cytochrome P450 genes *CYP6P9a* and *CYP6P9b*, were assessed by a quantitative reverse transcription PCR (qRT-PCR), as previously described (Kwiatkowska et al., 2013; Riveron et al., 2013). RNA (Rubonucleic acid) was extracted and purified using the picopure RNA isolation Kit (Life Technologies, Camarillo, CA, USA) according to the manufacturer's instructions. cDNA (complementary Deoxyribonucleic acid) was synthesized from the purified RNA by quantitative RT-PCR using the SuperScript III (Invitrogen, Waltham, MA, USA) and the oligo-dT20 and RNAse H (New England Biolabs, Ipswich, MA, USA) kit in a total reactional volume of 20 μL including of 19 μL PCR mix (10 μL of SyBr Green, 7.8 μL of dH2O, 0.6 μL of forward and reverse primers at the concentration of 10 mM for each gene of interest), and 1 μL of cDNA (or dH2O water for controls). Amplification was performed with an initial step of denaturation at 95 °C for 3 min followed by 40 cycles of 10 s at 95 °C, 10 s at 60 °C, then one cycle of 1 min at 95 °C, 30 s at 55 °C and 30 s at 95 °C. After normalization with housekeeping genes Actin (AFUN006819) and RSP7 (AFUN007153-RA), the relative expression for each gene was calculated according to the 2^-ΔΔCT^ method (Schmittgen and Livak, 2008). The level of significance in the gene was performed using unpaired Student *t-*test.

## 3 Results

### 3.1 The *CYP6P9b*_R resistant allele reduces the fecundity/fertility of female mosquitoes

**Fig. 1:**
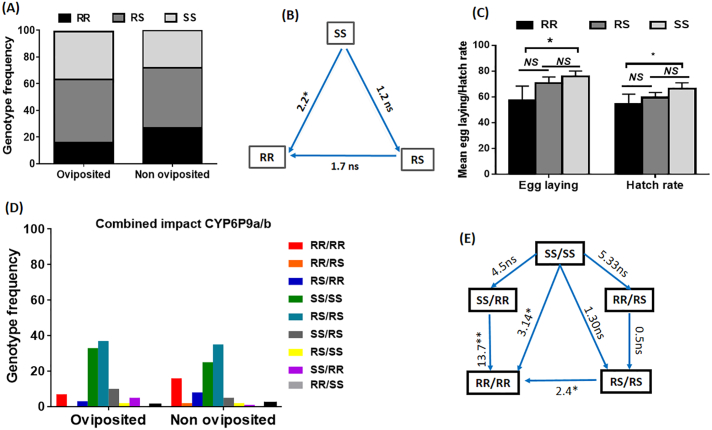
Impact of multiple P450 (*CYP6P9a* and *CYP6P9b)* genes on fecundity and fertility of females *An. funestus*: (A) and (B) Schematic representation of the impact of *CYP6P9b* genotypes on laying success with odd ratio (OR); (C) Comparison of the mean number of eggs laid and hatching rate between the three genotypes; (D) and (E) Schematic representation of the cumulative impact of *CYP6P9a* and *CYP6P9b* genotypes on laying success with odd ratio (OR). ***: significant difference at *p* < 0.001; * significant difference at *P* < 0,05; *NS*: not significant.

No significant difference (χ^2^ = 3.8; *p* = 0.1) was observed in the distribution of genotypes between females which have successfully laid eggs after blood feeding and those which did not lay eggs although a trend of a higher proportion of homozygote susceptible was observed in those that laid (Fig. 1A). Correlation analysis (using odds ratio (OR)) for oviposition between homozygote resistant mosquitoes (RR), homozygote susceptible (SS) and heterozygote mosquitoes (RS) with significance established using Fisher's exact probability test, revealed that SS mosquitoes have a greater ability to lay eggs than RR (OR = 2.2; confidence interval (CI) 95%: 1.0–4.8; *p* = 0.04) (Fig. 1B). No difference was observed between SS and heterozygote (RS) (OR = 1.2; CI 95%: 1.2–2.3; *p* = 0.3) (Fig. 1B). This suggests that mosquitoes harboring the *CYP6P9b*-R resistant allele have less chance to lay eggs compared to those with the susceptible allele. Mosquitoes with RS genotype displayed the same ability of oviposition than RR (OR = 1.7; CI 95%:0.8–3.7; *p* = 0.09) (Fig. 1B) suggesting a non-additional burden of the *CYP6P9b*_R allele on fecundity.

Furthermore, when analysing the quantity of eggs laid, females with *CYP6P9b*_SS genotype laid more eggs (76.0 ± 4.4) than RR (55.2 ± 11.2) (*p* = 0.007) but no significant difference was found between RS (70.8 ± 4.8) and RR (55.2 ± 11.2) (*p* = 0.09) (Fig. 1C). Concerning the viability of eggs laid (hatching rate), the mean number of larvae was lower in RR (38.6) compared to RS (43.4) and SS (55.15) (*p* = 0.03). Accordingly, the hatch rate also was lower in RR compared to other genotypes (*p* < 0.04) (Fig. 1C).

### 3.2 *CYP6P9b* combines with *CYP6P9a* to further reduce the fecundity of females *An. funestus*

As *CYP6P9b* was shown not to be necessarily linked to *CYP6P9a (*Mugenzi et al., *2019**)*, we next assessed how combinations of genotypes at both genes affect the fecundity of *An. funestus*. The independent segregation of genotypes at both genes was confirmed when analysing the influence of combined genotypes on the ability of females to lay eggs. Several combinations of genotypes were observed including RR/RR, RR/RS, RS/RS, SS/RS, and SS/SS (Fig. 1D). A comparison of the distribution of both sets of genotypes revealed that mosquitoes double homozygote resistant (RR/RR) at both genes had by far a significantly lower ability to lay eggs than all other combinations showing that the fitness cost induces by both genes acts additively to reduce the fecundity of females when mosquitoes are double homozygote resistant. Mosquitoes with RR/RR genotype had less chance for oviposition compared to SS/SS (OR = 3.1; CI = 1.1–8.4; *P* = 0.02) and to RS/RS (OR = 2.4; CI = 1.0–6.5; *P* = 0.05). The same trend was seen against other combinations although not significant; RR/RS (OR = 1.3; CI = 0.1–14.9; *P* = 0.6) and RS/SS (OR = 2.3; CI = 0.4–14; *P* = 0.3) (Fig. 1E).

### 3.3 Level of association between the *CYP6P9b*_R resistant allele and larval development time

**Fig. 2:**
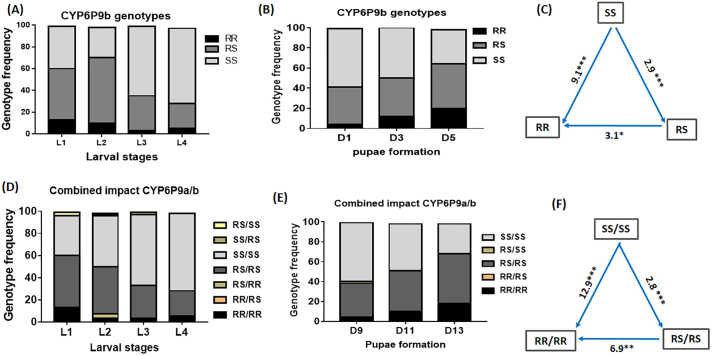
Influence of *CYP6P9b* and when combined with *CYP6P9a* on the development of immature stages. (A) Histogram of the variation in *CYP6P9b* genotypes frequency during the development of larvae (L1, L2, L3, and L4 represent different larval stages) and pupae formation (B); (C) and (D) represent the cumulative impact of *CYP6P9a* and *CYP6P9b* on the development of larvae and formation of pupae; (E) and (F) odd ratio of pupae formation for *CYP6P9b* only compared to cumulative impact of *CYP6P9a* and *CYP6P9b* showing that both markers combine to additively exacerbate the increase development time of double homozygote resistant (RR/RR) mosquitoes. ***significant difference at p < 0.001; ** significant difference at *p* < 0.01 and * significant difference at *P* < 0.05; *ns*: not significant.

On average, 100 randomly collected larvae for each L1, L2, L3, and L4 stages were genotyped for *CYP6P9b* to assess the influence of this marker on larval development. This revealed a significant and consistent decrease of the resistant allele *CYP6P9b*-R from L1 to L4 as noticed previously for *CYP6P9a*, indicating greater mortality or slower development of the resistant mosquitoes during this immature stage. The proportion of homozygote resistant RR decreases from L1 (11%) to L4 (5%) although this was not significant (χ^*2*^ = 1.7; *P* = 0.2) (Fig. 2A) probably due to the low number of mosquitoes with RR genotype. For the heterozygote RS genotype the decrease was significant from 49% in L1 to 27% in L4 (χ^*2*^ = 7.2; *P* = 0.007) together with a significant increase of the homozygote susceptible genotype SS from L1 (40%) to L4 (68%) (*χ*^*2*^ = 12.15; *P* = 0.0004). This highlights a significant fitness cost of *CYP6P9b* on the larval development of resistant mosquitoes.

Assessment of the rate of pupae formation by comparing the frequency of the *CYP6P9b* genotypes in the pupae obtained in D9 post-hatching, D11, and D13 showed an apparent decrease of the homozygote susceptible SS genotype from D9 (58%) to D11 (34%) (*χ* ^*2*^ = 1.73; *P* = 0.19) together with a significant increase of the homozygote resistant genotype RR and heterozygote RS from D9 to D13 (*χ* ^*2*^ = 11.17; *P* = 0.0008) confirming that homozygote susceptible mosquitoes developed significantly faster than homozygote resistant and heterozygote mosquitoes (Fig. 2B). Assessment of the OR for pupae formation further supported that *CYP6P9b*-SS mosquitoes developed significantly faster than *CYP6P9b*-RR (OR = 2.50; *p* < 0.01) whereas there was no difference with *CYP6P9b*-RS (OR = 1.18; *p* < 0.6) (Fig. 2C).

Furthermore, assessment of the cumulative impact of *CYP6P9a* and *CYP6P9b* on the time of development revealed a bigger impact than with CYP6P9b alone as the SS/SS genotype developed significantly much faster than RS/RS (OR = 2.8; *P* < 0.001) and RR/RR (OR = 12.9; P < 0.001). RS/RS developed also significantly faster than RR/RR (OR = 6.9; *P* < 0.01) (Fig. 2D-F; Table S2).

### 3.4 Assessment of the association between *CYP6P9b*-R allele and adult longevity

**Fig. 3:**
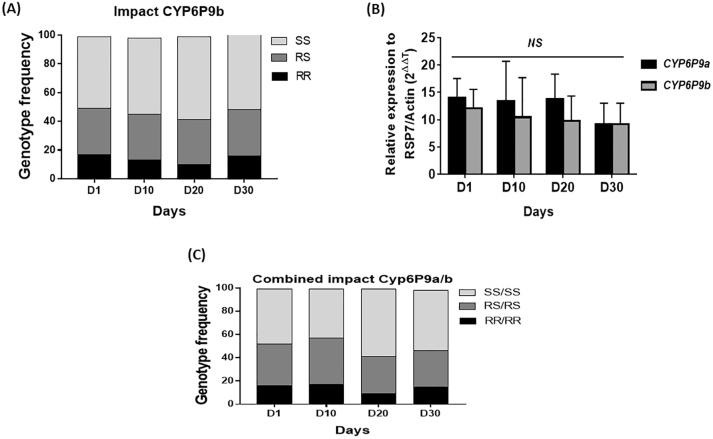
Influence of *CYP6P9b* and when combined with *CYP6P9a* on the adult longevity of *An. funestus*. (A) distribution of *CYP6P9b* genotypes at different time in the survived mosquitoes; (B) differential expression by quantitative reverse-transcription polymerase chain reaction of *CYP6P9a* and *CYP6P9b* genes in alive mosquitoes at different time points compared with the susceptible lab strain FANG. Error bars represent standard error of the mean; (C) cumulative effect of *CYP6P9a* and *CYP6P9b* on adult longevity showing no significant difference.

No difference was observed in the longevity of *CYP6P9b-*resistant mosquitoes compared to the susceptible as previously observed for *CYP6P9a*. After the genotyping of 100 alive mosquitoes at D1, D10, D20, and D30 after the adult emergence, the association between the *CYP6P9a*-R allele and adult longevity was assessed. Comparison of genotypes frequency showed no difference in the distribution of genotypes (χ^2^ = 2.7; *p* = 0.8) (Fig. 3A). In addition, assessment of the OR showed no difference in the life span of SS compared to RR (OR < 1.1; *p* > 0.4) and RS (OR < 1.1; *p* > 0.2). Evaluation of the expression level of *CYP6P9b* at the different time-points showed no significant difference in the level of expression of this gene (F = 1.08 df = 3; *p* = 0.4). The relative expression recorded was 12.03 ± 3.50 fold-change (FC) in D1, 10.4 ± 7.2 FC in D10, 9.7 ± 4.6 FC in D20 and 9.2 ± 3.8 FC in D30 (Fig. 3C). This suggests that over-expression of this P450 gene is not affecting the longevity of female mosquitoes. Furthermore, the combination of *CYP6P9a* and *CYP6P9b* genotypes did not also affect the longevity of females *An. funestus* (χ^*2*^ = 0.15; *P* = 0.7)*.*

### 3.5 Assessment of the reversal to susceptibility

**Fig. 4:**
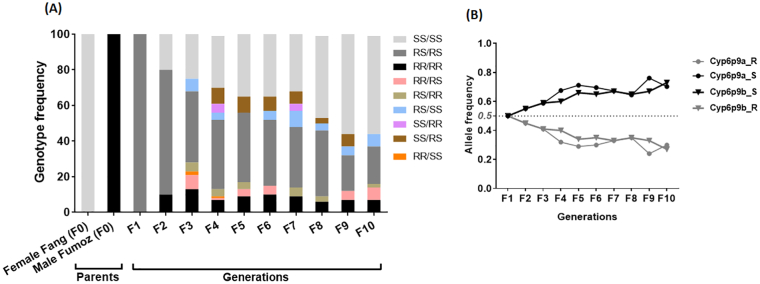
Evaluation of the reversal to susceptibility in the Hybrid colony Fang/Fumoz: Changes in the combined *Cyp6p9b* and *Cyp6p9a* genotypes (A) and allele (B) for ten generation in the insecticides free-environment. F represents each generation; Dotted line indicates a frequency of 50% for the resistant and susceptible alleles.

In cage experiments, a potential reversal to susceptibility was assessed by examining the changes in the frequency of *CYP6P9b*-R allele over 10 generations in the absence of insecticide selection pressure. A frequency of 50% of the resistant allele was confirmed in the F_1_ generation of the FANG/FUMOZ as well as a 50% for the susceptible allele. A significant and consistent increase in the proportion of homozygote susceptible mosquitoes was observed from F_2_ (20%) to F_10_ (58.1%) (χ^*2*^ = 7.8; *P* = 0.005) (Fig. S1) suggesting a reversal to susceptibility. Accordingly, an increase in the frequency of the susceptible allele was observed from F_2_ (50%) to F_10_ (73%) (χ^*2*^ = 5.9; *P* = 0.01) (Fig. S1; Table S1). Furthermore, an increased frequency of SS/SS double homozygote susceptible genotype was observed (χ^*2*^ = 6.3; *P* = 0.01) (Fig. 4A&B).

## 4 Discussion

In this study, using the recently designed DNA-based diagnostic assay for cytochrome P450 *CYP6P9b* gene, we investigated the fitness cost imposed by this P450-based resistance on various life-traits of *An. funestus*. This revealed a significant cost imposed by *CYP6P9b* and that this marker combines with *CYP6P9a* to additively exacerbate the fitness cost caused by pyrethroid resistance.

### 4.1 Influence of *CYP6P9*b metabolic resistance on the life traits of *An. funestus*

The *CYP6P9b* induced a reduction in mosquitoes' fecundity and fertility as previously observed for *CYP6P9a (*Tchouakui et al., *2020c**).* The proportion of females that laid eggs was lower in the resistant and heterozygote genotypes than for susceptible and females with resistant genotype *CYP6P9b*-RR showed a significantly reduced number of eggs and larvae compared to females with the *CYP6P9b*-SS susceptible genotype. Also, we observed a greater mortality/slower development of the resistant mosquitoes during larval development compared to the susceptible ones as previously observed for the *CYP6P9a*. Such greater mortality associated with slower development of mosquitoes with the *CYP6P9b-*R resistant allele could be linked to the fact that despite all the three genotypes were maintained in the same larval bowl, larvae with *CYP6P9b-*R resistant allele were probably less competitive for food and space compared to those with the susceptible allele and therefore, developed significantly slower. As observed previously in resistant *Culex pipiens* for carboxylesterase-mediated metabolic resistance (Foster et al., 2003), the over-expression of *CYP6P9a/b* is probably linked with a decreased locomotive performance limiting the ability of mosquitoes with the resistant allele to move faster to feed. All this together could explain the longer developmental time observed in *CYP6P9b*-RR homozygote resistant mosquitoes compared to heterozygotes *CYP6P9b*-RS and *CYP6P9b*-SS susceptible mosquitoes. However, in comparison with fecundity and larval development, there was no association between the *CYP6P9b*-R resistant allele and adult longevity similar to previous observation with *CYP6P9a*. This observation may suggest that the impact of *CYP6P9b*-R on the vectorial capacity of resistant mosquitoes might be less pronounced than that observed for the L119F-GSTe2 mutation which was shown to increase the longevity of resistant mosquitoes (Tchouakui et al., 2018a). However, this needs to be assessed in field conditions. Rivero *et al* reported that GSTs are known to protect mosquitoes against oxidative stress which results in increased longevity whereas the increased activity of monooxygenases is associated with increased oxidative stress in mosquitoes (de Montellano and De Voss, 2005). The increased oxidase stress due to the overproduction of monooxygenases could, therefore, reduce the longevity of insects although no such impact was seen in this study. Further studies with field populations will help further assess the extent of the effect of *CYP6P9b* gene on the lifespan of resistant mosquitoes in natural conditions.

### 4.2 *CYP6P9*b combines with *CYP6P9a* to exacerbate the fitness cost of pyrethroid resistance in *An. funestus*

In this study, mosquitoes that are double homozygotes resistant (RR/RR) at both *CYP6P9a* and *CYP6P9b* had by far, a significant low ability to lay eggs and slow development than mosquitoes with other combinations of genotype showing that both genes act additively to reduce the fecundity of double homozygote resistant female mosquitoes and reduced also the speed of development of immature stages. Reduction of the fecundity and slow development of larvae has been reported for other resistance mechanisms in dengue (Brito et al., 2013; Belinato et al., 2012; Martins et al., 2012b) and malaria (Tchouakui et al., 2020b; Tchouakui et al., 2018b) vectors. However, this is the first study evaluating the fitness cost of multiple cytochrome P450-based resistance. In the previous study with experimental hut assessing the impact of *CYP6P9b* beside *CYP6P9a,* it was noticed that *CYP6P9b* does independently reduce the efficacy of bed nets particularly pyrethroid-only nets beside *CYP6P9a*. In the same study, a greater reduction of bed net efficacy was observed when both *CYP6P9b* and *CYP6P9a* were combined since double homozygote resistant mosquitoes were by far more able to survive exposure to pyrethroid-only nets than all genotypes. Such an additive burden of the duplicated cytochrome P450s indicates the greater risk that metabolic resistance poses to insecticide-based interventions. This also supports the concern highlighted in the WHO global plan for insecticide resistance management that if nothing is done pyrethroid resistance could lead to an increased burden of malaria in Africa (WHO, 2012). However, a greater fitness cost imposes by *CYP6P9b* when combined with *CYP6P9a,* showed that if suitable resistance management strategies such as rotation were implemented early enough, P450-based resistance could be managed. This should encourage future strategies using non-pyrethroid-based LLINs to reduce the selection pressure and allow such rotation to slow the spread of pyrethroid resistance.

### 4.3 Reversal to susceptibility in insecticide-free environment

Knowledge of the reversal rate for insecticides such as pyrethroids is crucial before implementing any resistance management strategy in the field. As recently observed for *CYP6P9a*-R, in this study, a significant decrease in the frequency of the *CYP6P9b*-R resistant allele was observed after ten generations in the insecticide-free environment, which corresponds to around 1 year. Also, a significant increase in the frequency of SS/SS susceptible genotype was observed. This reduction in the resistant allele frequency could be attributed either to the accumulation of deleterious effects observed in some life traits of the vector as noticed for fecundity and larval development here (Saavedra-Rodriguez et al., 2012) or to the pleiotropic effect of other genes very close to *CYP6P9a* and *CYP6P9b*. Mating, copulation, and insemination efficiency are other key factors that were not assessed in this study but which could have contributed to the reversal observed since females anopheles are inseminated only once during their lifespan.

## 5 Conclusion

This study investigating the fitness cost of duplicated P450-based metabolic resistance to pyrethroids in *An. funestus* revealed that *CYP6P9b*_R resistance allele combines with the previously detected *CYP6P9a*_R to impose a significant fitness cost on fecundity, fertility, and larval development of resistant mosquitoes. This study highlights the greater fitness cost imposed by the over-expression of multiple P450s. It shows that if suitable resistance management strategies such as rotation were implemented at an early stage, P450-based resistance could be managed thus, future strategies using non-pyrethroid-based LLINs should be encouraged to reduce the selection pressure and allow such rotation to slow the spread of pyrethroid resistance.

## Author contributions

C.S·W. conceived and designed the study; Ma.T carried out the sample collection; Ma.T, D.D., Ma.T. reared and maintained the strain in the insectary; Ma.T. Mi.T, L.M.J.M. and M.J.W performed the molecular analyses; Ma.T, and C.S.W. analyzed the data; Ma.T and C.S.W. wrote the manuscript with contributions from F.N.. All authors read and approved the manuscript.

## Funding

This work was supported by the Wellcome Trust (Welcome senior 101,893/Z/13/Z and 217,188/Z/19/Z) awarded to CSW.

## Declaration of Competing Interest

The authors declare no conflicts of interest.

## Appendix A Supplementary data

Supplementary material Image 1
