# Supplementary material for: Combined over-expression of two cytochrome P450 genes exacerbates the fitness cost of pyrethroid resistance in the major African malaria vector Anopheles funestus
Source: Pestic Biochem Physiol. 2021 Mar;173:104772. doi: 10.1016/j.pestbp.2021.104772 (PMC8024743; doi:10.1016/j.pestbp.2021.104772)
Supplement: Supplementary file 1 — Supplementary material [file mmc1.docx]

**Supplementary file**s

**Table S1**: variation in the *CYP6P9b* genotypes and alleles frequency for ten generations in the insecticides free-environment

|  | **Generations** | | | | | | | | | |
| --- | --- | --- | --- | --- | --- | --- | --- | --- | --- | --- |
|  | ***CYP6P9b* genotypes** | | | | | | | | | |
|  | **F1** | **F2** | **F3** | **F4** | **F5** | **F6** | **F7** | **F8** | **F9** | **F10** |
| ***CYP6P9b*_RR** | 0  (0%) | 2  (10%) | 6  (15%) | 23  (9%) | 09  (15%) | 12  (12%) | 5  (10%) | 11  (12%) | 5  (09%) | 5  (14%) |
| ***CYP6P9b*_RS** | 45  (100%) | 14  (70%) | 21  (52%) | 68  (47%) | 40  (52%) | 46  (46%) | 26  (47%) | 44  (47%) | 19  (47%) | 13  (31%) |
| ***CYP6P9b*_SS** | 0  (0%) | 4 (20%) | 13  (32%) | 52  (44%) | 36  (32%) | 41  (41%) | 24  (43%) | 38  (40%) | 20  (44%) | 25  (55%) |
| **Total** | **45** | **20** | **40** | **137** | **85** | **99** | **55** | **93** | **44** | **43** |
|  | ***CYP6P9b* alleles** | | | | | | | | | |
| ***CYP6P9b*_R** | 50% | 45% | 41% | 40% | 34% | 35% | 33% | 35% | 33% | 27% |
| ***CYP6P9b*_S** | 50% | 55% | 59% | 60% | 66% | 65% | 67% | 65% | 67% | 73% |
| **Total** | **100%** | **100%** | **100%** | **100%** | **100%** | **100%** | **100%** | **100%** | **100%** | **100%** |

*F represents each generation

**Table S2:** variation in the frequency of combined genotypes at the *CYP6P9* and *CYP6P9b* locus during the larval development and the pupae formation

| **Combined genotypes at the *CY6P9a* and *CYP6P9b* locus** | **Larvae L1** | **Larvae L2** | **Larvae L3** | **Larvae L4** | **Pupae D1** | **Pupae D3** | **Pupae D5** |
| --- | --- | --- | --- | --- | --- | --- | --- |
| **RR/RR** | 13 (13.1%) | 2 (2.7%) | 3 (3.2%) | 4 (6.0%) | 2 (4.5%) | 5 (9.8%) | 11 (18.6%) |
| **RR/RS** | 0 (0%) | 1 (1.4%) | 0 (0%) | 0 (0%) | 0 (0%) | 1 (2.0%) | 0 (0%) |
| **RS/RR** | 0 (0%) | 5 (4.1%) | 0 (0%) | 0 (0%) | 0 (0%) | 0 (0%) | 0 (0%) |
| **SS/SS** | 39 (39.4%) | 20 (46.6%) | 60 (64.5%) | 47 (70.1%) | 26 (59.1%) | 14 (27.4%) | 18 (30.5%) |
| **RS/RS** | 47 (47.5%) | 29 (43.8%) | 28 (30.2%) | 16 (23.9%) | 15 (34.1%) | 21 (41.2%) | 30 (50.9%) |
| **SS/RS** | 0 (0%) | 15 (1.4%) | 2 (2.1%) | 0 (0%) | 0 (0%) | 10 (19.6%) | 0 (0%) |
| **RS/SS** | 0 (%) | 1 (1.4%) | 0 (0%) | 0 (0%) | 1 (2.3%) | 0 (0%) | 0 (0%) |
| **Total** | **99 (100%)** | **73 (100%)** | **93 (100%)** | **67 (100%)** | **44 (100%)** | **51 (100%)** | **59 (100%)** |

**Figure S1. Evaluation of the reversal to susceptibility in the Hybrid colony Fang/Fumoz:** Changes in the *Cyp6p9b* genotypes **(A)** and allele **(B)** for ten generations in the insecticides free-environment. F represents each generation; Dotted line indicates a frequency of 50% for the resistant and susceptible alleles

**
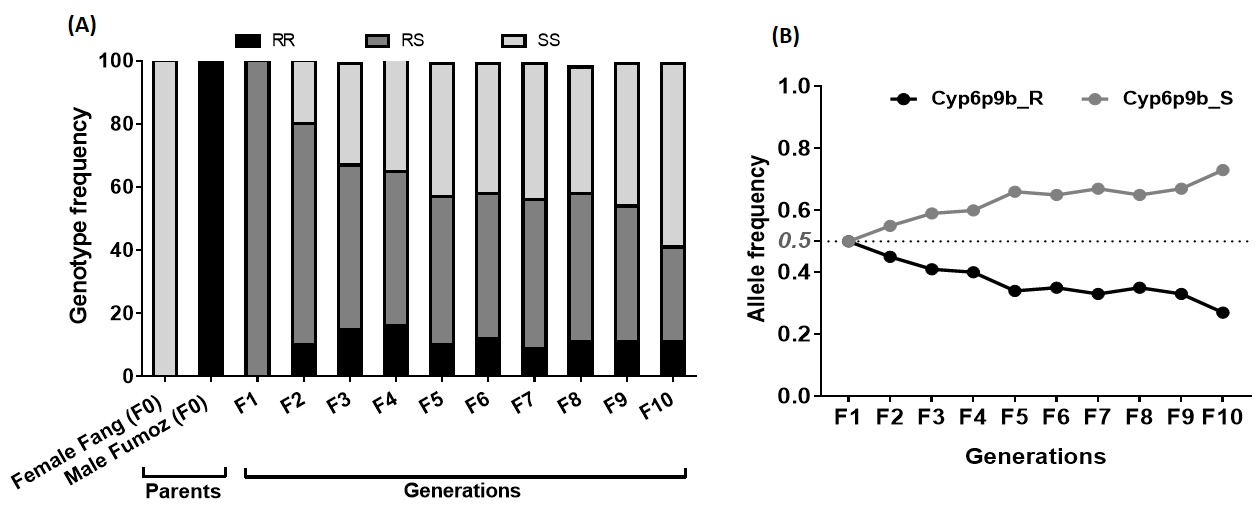
**
